# The Potential of Long-Acting, Tissue-Targeted Synthetic Nanotherapy for Delivery of Antiviral Therapy Against HIV Infection

**DOI:** 10.3390/v12040412

**Published:** 2020-04-07

**Authors:** Anna Halling Folkmar Andersen, Martin Tolstrup

**Affiliations:** 1 Department of Infectious Diseases, Aarhus University Hospital, 8200 Aarhus, Denmark; mtol@clin.au.dk; 2 Department of Clinical Medicine, Aarhus University, 8000 Aarhus, Denmark

**Keywords:** HIV, drug delivery, antiretroviral therapy, HIV reservoir, nanotherapy

## Abstract

Oral administration of a combination of two or three antiretroviral drugs (cART) has transformed HIV from a life-threatening disease to a manageable infection. However, as the discontinuation of therapy leads to virus rebound in plasma within weeks, it is evident that, despite daily pill intake, the treatment is unable to clear the infection from the body. Furthermore, as cART drugs exhibit a much lower concentration in key HIV residual tissues, such as the brain and lymph nodes, there is a rationale for the development of drugs with enhanced tissue penetration. In addition, the treatment, with combinations of multiple different antiviral drugs that display different pharmacokinetic profiles, requires a strict dosing regimen to avoid the emergence of drug-resistant viral strains. An intriguing opportunity lies within the development of long-acting, synthetic scaffolds for delivering cART. These scaffolds can be designed with the goal to reduce the frequency of dosing and furthermore, hold the possibility of potential targeting to key HIV residual sites. Moreover, the synthesis of combinations of therapy as one molecule could unify the pharmacokinetic profiles of different antiviral drugs, thereby eliminating the consequences of sub-therapeutic concentrations. This review discusses the recent progress in the development of long-acting and tissue-targeted therapies against HIV for the delivery of direct antivirals, and examines how such developments fit in the context of exploring HIV cure strategies.

## 1. Introduction

Combination antiretroviral therapy (cART) has transformed HIV from a life-threatening disease to a manageable infection. Treatment-adherent individuals with full viral suppression can live healthy, long lives and are unable to transmit the virus to others. Yet, in 2018, there were still approximately 770,000 AIDS-related deaths and 1.7 million people became infected with HIV [1]. Even though antiretroviral drugs (ARVs) have the ability to decrease plasma viral load to below detection, these drugs are not able to clear all viruses from the host. It is therefore evident that despite huge success in the development of treatment and prevention, a cure is still warranted.

The formation of latent viral reservoirs that persist despite having none to very limited viral replication prevents a successful cure with suppressive cART. These viral reservoirs can be defined on the basis of the cells that are infected e.g., infected T- or myeloid cells, or the anatomical sites such as lymph nodes (LN), central nervous system or gut-associated lymphoid tissues (GALT), where these cells reside [2]. A recent report from studies of simian immunodeficiency virus (SIV)-infected macaques suggests that 98.6% of SIV vRNA+ cells reside in lymphatic tissues (i.e., LNs, spleen, GALT) [3].

The particular biology of infected cells and limited tissue penetration of ARVs in concurrence may cause the presence of a viral reservoir [4,5,6]. Independent of mechanism, a cure for HIV would entail targeting the major sites where these viral reservoirs persist. The “shock and kill” approach is one of the strategies pursued to achieve an HIV cure. An important component of this approach is the use of a latency-reversing agent (LRA) that shocks the virus into a more active form. Consequently, the activated virus would then be rendered susceptible for clearance by the immune system [7].

In most cases, a cure approach would be accompanied by cART to prevent the infection of new cells. Many of the compounds available today suffer from short half-lives and limited tissue penetrations, which consequently could impede their activity at the most relevant viral reservoir sites [8]. Moving forward, it may be of importance to study and optimize both existing and newly developed ARVs as well as the components of HIV cure treatments for enhanced viral reservoir targeting/penetrance.

This review discusses the potential of long-acting, tissue-targeting synthetic scaffolds that enable delivery of cART and HIV cure agents to HIV-burdened lymphatic tissues. Please refer to other excellent reviews for details on the penetration of nanotherapeutics into the central nervous system [9,10]. Particularly, this review will focus on how features from nanotechnology can aid in overcoming some of the key challenges faced by both cART and HIV cure strategies, including low bioavailability, low tissue penetration in lymphatic tissues and short plasma half-lives.

## 2. State of the ART

Strict adherence and continuous viral monitoring have conditioned HIV+ people to live long lives. The current treatment consists of a set combination of two or three ARVs that must be administered daily. Because of the high mutation rate of the HIV genome during viral replication, monotherapy quickly results in the emergence of drug-resistant viral strains and treatment failure. Inhibiting multiple different enzymes and blocking important host–virus interactions in the viral life cycle are key to fully block viral replication and prevent resistance development. However, upon cessation of cART, the virus will rebound and become detectable in plasma typically within weeks [11]. Several classes of antiretroviral drugs have been developed and are grouped based on which step of the HIV replication cycle they inhibit. Overall, the different drug classes are: (1) nucleoside and non-nucleoside reverse transcriptase inhibitors (NRTIs and NNRTIs, respectively), (2) protease inhibitors (PIs), (3) fusion inhibitors, (4) entry inhibitors and (5) integrase inhibitors (INIs) [12]. The different currently marketed pharmaceuticals are presented in Table 1. To fully comprehend what determines the dose effectiveness of treatment, one needs to understand the sequential barriers and sites of loss that orally administered antiretrovirals face before exerting their intended antiretroviral function. This following section will elucidate the path from administration to cellular function of current ARVs.

## 3. Bioavailability and Pharmacokinetics

The first challenge of orally administered drugs is absorption through the gut wall. Because many ARVs, particularly protease inhibitors, are poorly water-soluble, incomplete dissolution in the gut lumen decreases transfer across the intestine wall [51]. Consequently, a large fraction of the ingested compound never reaches systemic circulation, which is often the main factor for a low plasma bioavailability [52]. Table 1 lists the calculated bioavailability of different ARVs.

Once inside the lamina propria of the gut, a minor fraction of the drug will be drained by the lymph capillaries but the majority will be transported by blood vessels that drain the interstitial space to the portal vein that passes through the liver. The liver is responsible for the first-pass metabolism of many compounds, including ARVs. Thus, ARVs are partially metabolized and cleared from the blood system before even reaching systemic circulation. The fraction that does leave the liver intact goes through the vena porta to the heart and lung system before being dispersed into the arterial system. In this blood system, certain ARVs suffer from a short terminal plasma half-life (T_½_) (listed in Table 1). Short plasma half-life is caused by a combination of multiple factors, including size and kidney excretion, and other physicochemical properties such as high hydrophobicity or high affinity for metabolizing enzymes in the liver. A particularly classic example is the PI, saquinavir, a very hydrophobic molecule with high substrate affinity for the efflux transporter P-glycoprotein (P-gp) and the hepatic enzyme, cytochrome P4505A (CYP3A). This results in efficient transport out of the cells and a rapid metabolism in the liver and subsequent excretion of saquinavir [53,54]. Consequently, the bioavailability of saquinavir following oral dosing is only 4% of the administered dose. Therefore, the clinical application of saquinavir is lower due to an increased risk of hepatic side effects at therapeutic doses [55]. These factors are accounted for in the dosing regimen; however, the risk of reaching sub-therapeutic concentrations with a missed or delayed dose is increased.

## 4. Tissue Penetration

The relatively high permeability of lymph capillaries in the peripheral tissues such as the lamina propria of the gut causes the lymph capillaries and lamina propria to be similar in composition on many parameters. However, ARVs can be excluded from entering the lymphatic system directly because the equilibrium between lymph and interstitial space is affected differently by physicochemical properties as well as a low flow rate in the draining lymph capillaries [4]. Properties of compounds that are associated with a higher degree of lymphatic uptake include higher molecular weight, higher overall particle size and higher lipophilicity [56,57,58,59]. The resulting limited tissue penetration of ARVs leads to sub-therapeutic concentrations in many important virological sites such as the lymphatic tissues, particularly the lymph nodes. Whether this can facilitate continuous low-level HIV replication in these tissues and contribute to the rapid rebound seen upon cessation of cART has been speculated [6,60,61].

In summary, there are several major challenges associated with the current approved pharmaceuticals against HIV infection. The overall aim of rethinking cART and cure agents in the context of nanotherapeutics is to have the physicochemical properties of the drug delivery platform dictate the fate of the antivirals in vivo [62,63].

## 5. What are Nanotherapeutics for HIV Treatment and Cure?

Combining antiretroviral drugs with a synthetic molecule can be an attractive approach to solve some of the inherent challenges with the small molecule drug itself. Because these synthetic compounds are typically in the 1–100 nanometer range, they are referred to as nanotherapeutics. Various types of nanotherapeutics have already been developed for other healthcare applications, particularly cancer therapy, where a compound such as PEG-lipidated Doxorubicin (Doxil [64]) is used as a first-line therapy against a range of solids tumors e.g., breast and ovarian cancer [65,66,67].

**Figure 1:**
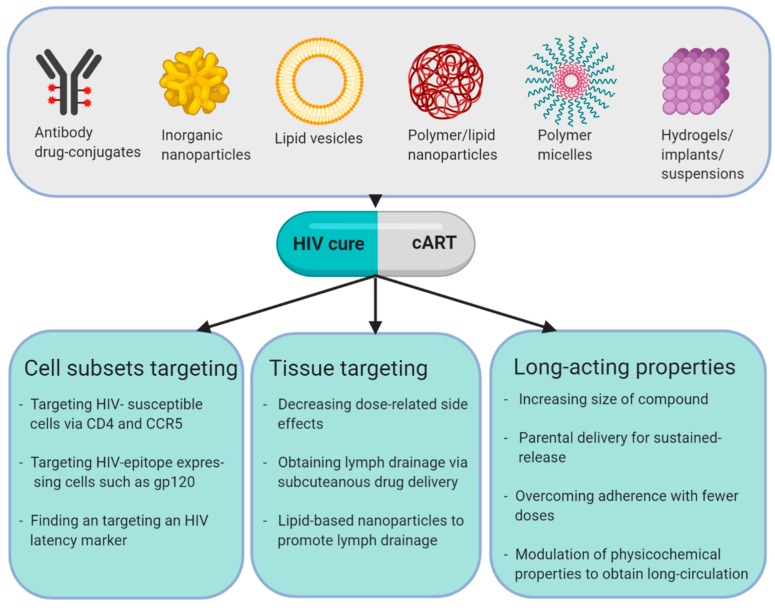
Examples of types of nanotherapy and their application in HIV treatment and cure. Created with Biorender.com.

Many different classes of synthetic molecules have been utilized as the nanotherapeutic scaffold for antiretroviral drug delivery, including nanoparticles comprised of polymers, lipids, liposomes, micelles, dendrimers and inorganic particles, e.g., gold nanoparticles [68]. Protein-conjugates, such as antibody–drug conjugates or albumin-conjugated compounds are sometimes also included when discussing nanotherapeutic compounds. In the interest of limiting the scope, this review discusses classical examples of these different synthetic scaffolds. The major synthetic strategies and the commonly associated benefits and of these different approaches are detailed in Figure 1. Antibody drug-conjugates have been extensively used for targeting e.g., CD4 for anti-HIV applications. Here, cells that do not express the antibody target are bypassed, and only cells bearing the target are engaged. In principle, antibodies can be added to most nanotherapeutic molecules as a targeting moiety [69]. Inorganic nanoparticles can be constructed by e.g., gold or silver nanoparticles and can have beneficial properties for anti-HIV drug delivery [70]. They can be internalized in different cell types such as macrophages, which can be either a benefit or a caveat depending on whether these were the intended target [68]. Their typical smaller size (compared to other nanotherapeutics) facilitate entry into tissues such as the central nervous system but also distinct pharmacokinetic profiles. Lipid vesicles were the first nanomaterial utilized for drug delivery. Lipid vesicles are closed, self-assembling spheres comprised of a bilayer of phospholipids. The lipid bilayer surrounds an aqueous space, wherein drugs are enclosed. These molecules can vary greatly based on preparation, their overall lipid composition, size and surface charge, and all of these parameters can influence biological effects and pharmacokinetics. The particles interact with cell surfaces for their drug delivery, either through adsorption, fusion of membranes or endocytosis. Disadvantages to lipid vesicles include their limited drug-loading capacities of hydrophilic drugs [71]. Polymer and lipid nanoparticles are heterogeneous in composition, size and overall shape. They can be comprised of biostable or biodegradable monomers and can carry drug agents via one of several mechanisms: chemically linked to the particle; entrapped or encapsulated within the polymer–lipid particle; or adsorbed onto the particle surface via hydrophobic interactions [72,73]. Polymer micelles consist of a hydrophobic core and a hydrophilic shell, forming a spherical micelle. Similarly, to the lipid vesicles, these structures can transport cargo within the central core, or in/on the surface of the shell. In addition, the micelles can be modified to include targeting moiety or other ligands that enable more specific drug delivery [73]. Another benefit of polymer systems such as PEG is that the overall structure becomes “masked” from immune recognition by preventing opsonization [74]. Hydrogel, implants and suspensions are a heterogeneous class of drug delivery modalities spanning from simple saline suspensions of nanotherapeutic drugs to gels and implants. Examples are suspensions of otherwise poorly soluble drugs with stabilizers (i.e., a surfactant) into nano-sized crystals to improve the overall solubility. These solutions have slow dissolution, facilitating sustained release into the blood after e.g., subcutaneous or intramuscular drug delivery. One potential caveat of these crystals in solution is that once in plasma they can be rapidly phagocytosed by macrophages e.g., Kuppfer cells in the liver [75]. Other examples in this group are polymers formulated in gel-form for local topical application, e.g., for protection of mucus barriers during intercourse, and injected/implantable, degradable devices that release drugs over time [76]. Importantly, many nanotherapeutic systems are combinations of these different scaffold classes.

All of these compounds are relatively biologically inert structures able either to encapsulate or to carry small molecule drugs through direct covalent linkage. However, the physicochemical and biologic properties of the nanotherapeutics can be modulated by the addition of either polyanionic, hydrophobic or hydrophilic side chains on the scaffold. Moreover, the compound can be further modified by the addition of targeting ligands [49]. Indeed, the construction design of the lipid or polymer scaffold is virtually unlimited, but some practical considerations must be taken into account. For example, in many current spherical non-covalent nanoparticles of physiological relevant sizes, the pharmaceutical active drugs comprise only 1% of the total molecular weight [49].

## 6. Long-acting Antiviral Prodrugs

Less than daily administration of effective cART is eminent, and the terminology of long-acting is currently defined as less-than-daily dosing. This description covers both new formulations of small molecule drugs into long-acting oral formats and the field of nanotherapeutics. The latter is sometimes referred to as sustained-release, extended-release or controlled-release, but covers the same meaning: a novel compound, drug-delivery platform or formulation whose physicochemical properties ensures long-circulation and thus long-acting antiretroviral therapy. Much attention has been put on parental drug delivery over oral [76].

Currently, two phase III clinical trials are investigating intramuscular delivery of a polymer-based, long-acting formulation of cabotegravir and rilpivirine (ATLAS (NCT02951052) and FLAIR (NCT02938520)) [77]. The plasma half-life of the integrase inhibitor cabotegravir is 40 h after oral administration [78] and rilpivirine, an NNRTI, halves in 48 h. This combination has promising use as a once-monthly maintenance therapy after an oral lead-in phase. Despite the therapy being well tolerated, the US FDA declined the approval of this treatment regimen in December 2019. The reasons for rejection were regarding chemistry, manufacturing and controls (CMC) [77,78,79].

Other preclinical studies aiming to identify long-acting small anti-HIV molecules have been published. For example, Yant et al. identified a novel, potent capsid inhibitor, GS-CA1 displaying a long plasma half-life after subcutaneous (s.c.) delivery [80]. Because this compound is highly insoluble in water, it relies on the slow diffusion from the initial depot injection in the interstitial space for sustained activity. However, despite impressive half-life data and high anti-viral efficacy, no evidence of improved drug-delivery to HIV reservoirs was given and the drug was not tested as part of a cART regimen [80].

Increasing the size of a molecule by use of a synthetic polymer scaffold is another approach to extend its overall plasma half-life, caused by a size-dependent retention by the kidney excretion system. This is easily obtainable via nanotherapeutic approaches. Recent progress in polymer production strategies has enabled polymer synthesis to become a tightly controlled process, where polymer structures with low polydispersity can be created. Previously, polymer nanotherapeutics were impeded by unacceptable batch-to-batch variations as well as within-batch size heterogeneity. Combined with novel linker chemistry, for example self-immolative linkers (SIL), both polymer size and complexity as well as triggered release of drug can be controlled, yielding a very attractive drug-delivery strategy [81]. Some of the most commonly used polymer systems are poly(hydroxypropyl methacrylamide) (PHPMA) [82,83,84,85,86,87], poly(lactic-*co*-glycolic acid) (PLGA) [88], poly(methacrylates) (PMA) [89,90] and poly(ethylene glycol) (PEG) [91]. The benefit of combining direct conjugation to a polymer via linker chemistry is the possibility to co-deliver different pharmaceutical drugs on the same polymer scaffold. This type of design thus enables combination therapy with precise drug ratios that are specifically released upon intracellular stimuli—highly appealing for cART, potentially in combination with an HIV cure agent.

Our group sought to develop and utilize these features of polymers. We have focused on combining antiviral small molecule drugs with synthetic polymers in combination with lipids such as DSPE or endogenous proteins like albumin to develop long-acting drug-delivery platforms [81,82,83,84,85,86,87,92]. Because albumin has an incredible long circulation time in vivo in humans (i.e., 19–21 days [93]), it can be used to traffic compounds through circulation. However, direct albumin–drug conjugation can be complicated by both formulation, biocompatibility and storage challenges. Using a lipid-polymer, such as 1,2-distearoyl-sn-glycero-3-phosphoethanolamine poly(ethylene glycol) (DSPE-PEG) with a natural affinity for albumin in vivo can solve this [94,95]. Upon administration, the lipid will quickly bind to albumin and become trafficked by the protein through the body. Synthetic polymers such as PEG serve as the linker between the small molecule drug and the lipid [81].

Li et al. developed a structure based on a biodegradable nanoparticle of PEG-PLA (poly(ethylene glycol)-poly(lactic acid)) that encapsulated an HIV fusion inhibitor, T1144, and an experimental NNRTI, DAAN-14f. The compound displayed an improved pharmacokinetic profile over pristine drugs in rats after intravenous dosing without loss of anti-HIV efficacy in vitro [96]. Mandal et al. pursued a similar strategy with nanoparticles of PLGA encapsulating elvitegravir (EVG), emtricitabine (FTC) and tenofovir alafenamide (TAF), and found that this combination had HIV prevention effects in humanized mice [97,98,99].

Long-acting or sustained-release formulations can also be obtaining by utilizing the phagocytic features of monocyte-macrophages as a reservoir for nanoparticles [100,101]. After cellular uptake, these particles will slowly degrade and thus release ARVs. Recently, an interesting strategy was developed comprising a “long-acting-, slow-effective release antiretroviral therapy” strategy (Laser ART) that enabled delivery of ARVs such as dolutegravir, lamivudine, abacavir and rilpivirine encapsulated in lipid nanoparticles via myristoylation. The result was hydrophobic, lipophilic “nanocrystals”, which were non-specifically internalized into monocytes and macrophages, and slowly released to the surrounding extracellular space [102,103,104,105]. Interestingly, two out of seven humanized HIV-infected mice were cured when Laser ART was combined with CRISPR/Cas9-mediated excision of HIV DNA [106]. These mice did not have detectable HIV in blood, lymphoid tissues, bone marrow, spleen or brain five weeks after end of therapy.

In summary, these studies prove that using nanotherapeutic formulation techniques, it is possible to develop compounds that challenge the “once-daily”-paradigm. However, issues remain regarding the depot in case of adverse events, since stopping therapy and removing the compound from the body can pose a significant hurdle or even be impossible.

## 7. Increasing Access of Drugs to Lymphoid Tissues

Nanotherapeutics have proven particularly useful within cancer research, where the enhanced permeability and retention (EPR) effect facilitates large molecules to passively target tissues with higher blood supply and metabolism, such as solid tumors [107]. Despite this simple assumption not being directly translatable to HIV-burdened tissues, the knowledge acquired from the development of the technology can be applied. The lymph nodes are less permeable for cART than other tissues which means that the concentration of cART is lower in these tissues than in the peripheral blood [4]. Increasing drug penetration using nanotherapeutic compounds could help decrease the risk of de novo cell infections and potentially improve the potency of new HIV cures aiming to clear or control these viral reservoir tissues.

Access to the lymphatic system can be approached at different levels: Overall specific drug delivery to a target can be executed at either the level of the whole tissue or at the cellular level [108]. The term “targeting” dictates that a drug targets a specific molecule or specific biological pathway (e.g., CD4-targeting). The term “localization” specifies a drug with properties that enable higher concentration in a biological tissue due to either specific or unspecific properties.

Delivering drugs more directly to the HIV reservoirs would be of highest relevance. Because the peripheral lymph system is a one-way system for fluid and protein drainage from the interstitial space, subcutaneous drug delivery and via the lymph system could be of use to reach HIV lymphatic reservoirs.

So why are ARVs not administered subcutaneously similar to e.g., insulin? The lymph system drains directly into the systemic circulation, so slow release into the blood stream could be achieved, and decreased bioavailability caused by impaired mucosal uptake avoided. Delivery of ARVs through the lymphatic system would also benefit from bypassing first-pass elimination in the liver [56].

**Figure 2:**
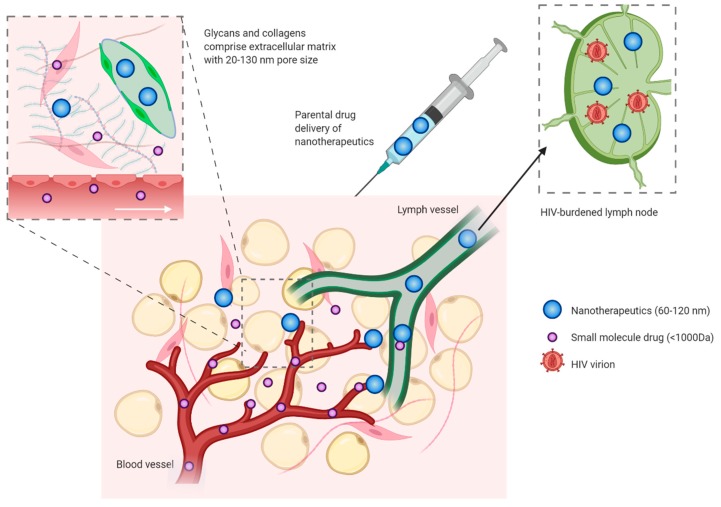
Interstitial space draining of nanotherapeutics. After subcutaneous injection of the nanotherapeutic particles, they will be dispersed in the interstitial space. This space is comprised of cells and an extracellular matrix, composed of glycans and collagens that will allow free passage of smaller molecules [63]. Based on size and physicochemical properties, such as lipophilicity, nanoparticles can either be removed via the draining venules or the peripheral lymphatic capillaries. Small molecule drugs are preferentially cleared via the draining blood vessels [56,109]. Created with Biorender.com.

Indeed, the initial lymph capillaries have free permeability to small molecules and these can thus diffuse unhindered through the initial lymph drainage system [63]. This causes the protein composition of intestinal fluids and lymph to be almost identical. Most of the fluid that will initially enter the lymph capillaries is resorbed in post-capillary venules and only larger molecules are retained in the lymph capillaries. The majority of molecules smaller than 10 nm such as ARVs are preferably absorbed from the interstitial space into post-capillary venules, whereas particles between 10 and 100 nm and above 16 kDa are efficiently removed from the interstitial space through the lymphatic system [56,109]. In addition, the drainage of fluids in the lymph capillaries is also significantly slower than drainage of fluids through the blood capillaries, which causes the majority of ARVs to pass directly into the blood and they are thus unaffected by the drug retention associated with the lymphatic system (Figure 2). Finally, co-dissolving multiple hydrophobic drugs like ARVs may result in inaccurate dosing due to aggregation or precipitation.

Along those lines, much attention has been given to delivery of modified lymph-compatible, nanotherapeutic ARVs that bypass the gut via parental administration. Contrasting to the protein composition between interstitial space and lymph capillaries, the lipid composition differs between these compartments because fats are readily resorbed into lymph [56,57]. This is relevant for lipid-based nanotherapeutic ARV delivery, based on subcutaneous administration. For example, lymphatic tissue localization of lipid-indinavir (IDV) particles via a passive diffusion strategy were explored [110,111,112,113,114]. IDV-particles were tested for antiviral (SIV) effects in non-human primates (NHPs) [110,111]. The results showed that 100 nm nanoparticles had optimal passive targeting abilities towards the lymphatic system, including the lymph nodes. Overall, the nanoparticles comprising the lipid-indinavir (IDV) complex resulted in up to 23-fold higher concentration of IDV in peripheral and visceral LNs [110].

The mechanism causing the biodistribution of nanoparticles in the lymphatic system have been investigated [115]. Specifically, it was shown that nanoparticles administered subcutaneously were retained intra-lymphatically and subsequently distributed to the entire mouse body [114,115]. This effect was ascribed to the relatively large size of the molecules (60–120 nm) preventing extravasation from lymph vessels, which blocks release into the blood stream and enables transport from lymph node to lymph node through interconnected lymphatic vessels [115] (such effect shown in Figure 2). In addition, the same research group have shown anti-HIV efficacy of a lipid formulation of three ARVs that alone have different solubilities (lopinavir, ritonavir and tenofovir). This combination was dubbed “the targeted, long-acting and combination anti-retroviral therapy” (TLC-ART). The main findings were enhanced and showed persistent drug levels throughout the body of NHPs for over a week [114], and importantly not exclusively in the draining, sentinel lymph nodes [110,112,116]. However, more research is needed in order to fully understand how compounds are retained intra-lymphatically.

## 8. Cellular Targeting

Particularly intriguing for HIV cure therapy is the direct targeting of compounds to specific cell types to achieve an increased drug concentration at certain anatomical locations. Targeting only cells that harbor virus or have a higher likelihood of being HIV-infected could reduce the off-target effects of the drug [62].

In theory, using a molecule with an affinity for a specific cell surface epitope could aid in drugs only interacting with the desired target. The HIV receptor, CD4, and co-receptor CCR5 have both been investigated for the purpose of delivering ARV [117]. Yang et al. attempted to deliver antiretroviral therapy in HIV-susceptible SUP-T1 lymphoblasts via anti-CD4-antibody-coated nanoparticles containing saquinavir [118]. They observed a two-fold increase in intracellular saquinavir concentrations. A similar approach, but with anti-CD4 antibody-coated liposomes with nevirapine and saquinavir, has also been explored [119]. Another strategy has also been tested with the direct conjugation of bryostatin (a protein kinase C activator) to anti-CD4 antibodies for reversing HIV latency in resting CD4+ T cells. In this study, they found that it was possible to increase activation of CD4 cells when targeting the nanoparticles to the CD4 receptor [120]. Lastly, a different strategy used fragments of gp120 conjugated to nanoparticles for achieving drug delivery of indinavir to HIV-infected CD4+ cells to increase the efficacy of the drugs [121,122].

Common for these targeting studies is minimal improvements in efficacy over native drugs. Missing is the evidence of actual intracellular uptake, to disprove that drugs are released extracellularly after binding to target via linker degradation. In fact, the CD4 molecule does not readily internalize, and this could compromise CD4 targeting as a feasible strategy [123,124]. Conversely, HIV peptides present on infected cells could also be the target. However, upon HIV infection, resting cells display limited expression of gp120 and other HIV proteins, which results in reduced viral targets for drug delivery. Despite this fact, efforts were made by Pollock et al. to deliver NB-DNF (an inhibitor of HIV gp120 folding) via liposomes coated with CD4 molecules to gp120-expressing cells [125]. The study showed the efficient viral inhibition of different HIV laboratory strains with up to 10^5^-fold increase in potency compared to free NB-DNF. The cells in this study were acutely infected with HIV, thus not a clear reflection of the clinical setting with years of HIV infection and latency.

A third and perhaps ideal, sophisticated approach to cellular targeting is to find a cell-derived epitope, only expressed on HIV-infected cells. A great deal of work has searched for a surface marker that could define an HIV-infected cell. Among such markers, CD32a was suggested to be preferentially expressed on cells harboring replication-competent HIV [126,127]. However, this finding was questioned by other research groups [128,129,130,131,132]. Additionally, two reports have investigated CD30 as a marker on CD4+ T cells which may be an interesting target [133,134]. These works highlight the importance, if possible, to define a reliable marker of HIV infection, but clearly continued research in basic cell biology and virology is needed in order to determine the feasibility of such approach.

## 9. Nanotherapeutics in an HIV-Cure Context

As briefly mentioned, the predominant strategy pursued in clinics (the shock and kill approach) may also benefit from modifications in drug delivery or formulation. LRAs such as histone deacetylase inhibitors (HDAC inhibitors) are widely used to reactivate latently infected cells [135]. During LRA treatment, cells are prevented from spreading HIV due to the presence of cART.

Nanotherapy could have a major role to play in this two-arm strategy. LRAs, like ARVs, are a group of mostly small molecule compounds (<1000 Da), and but diverse physicochemical properties and biological targets, and could thus similarly benefit from novel formulations strategies. Our group and others have attempted to synthetize LRAs in nanoparticles [86,87]. Following polymer linking of panobinostat (an LRA), we observed HIV-latency reversal in two latently infected cell lines, ACH2 (T cells) and U1 (monocytes). Thus, it is possible to develop polymer molecules conjugated with LRAs through SILs to achieve intracellular effects. Further, polymers and lipid-structured nanoparticles have been used to deliver LRAs in combination with direct ARVs. Nelfinavir, a protease inhibitor and bryostatin-2, was formulated into a lipid nanoparticle in the study by Kovochich et al. [120]. This enabled the reactivation and control of viral spreading in human T cell lines and latently infected cells isolated from humanized mice. Another preclinical study has shown a nanoparticle combination of vorinostat and tenofovir that was effective in the reactivation and suppression of HIV replication for five days in human astrocytes [136]. Further, Tang et al. used anti-CD45RO single chain variable fragments (scFv) of antibodies (targeting memory T cells) to direct a PLGA-PEG nanoparticle with vorinostat and nelfinavir to target cells harboring virus, while preventing infection of new cells [137]. They found binding of the memory T cells and blockage of the production of new virions.

In summary, these pioneering studies elucidate the creativity as well as the broad flexibility found within nanotherapeutic design and synthesis, which can be used to both target, reverse latency and deliver cART.

## 10. Future Directions

A treatment able to completely clear or control HIV in patients could have huge commercial prospects. The use of nanotechnology within pharma has already influenced the development of novel therapeutic compounds, especially for cancer treatment. The overall conclusion is that within nanotechnology lies opportunities for the development of compounds that use known and safe ARVs in new formulations that could aid in overcoming some of the shortcomings of current HIV treatment. This includes aims to (1) increase drug solubility, (2) extend plasma half-life and bioavailability, (3) targeted to- and enhanced retention in HIV-burdened tissues and cells, (4) deliver precise ratios of combination therapy leading to synergistic effects. In addition, the adaptation of HIV cure drugs to biocompatible nanotherapeutic scaffolds could aid in improved biodistribution, pharmacokinetic profiles and tissue targeting, as well as decreasing dose-related toxicities. Moreover, modeling the ratios of different therapeutics with different biological targets on a scaffold could reveal novel synergistic combinations by ensuring that drugs were delivered in precise amounts to the target cell or organ. A sterilizing cure for HIV has become imaginable with the advancement of gene therapy. This includes complete removal of the provirus by e.g., CRISPR-Cas9-mediated excision. Utilizing a nanotherapeutic drug delivery approach to tissues could potential overcome problems with both delivery of guide RNAs as well as reduce off-target effects [138].

The application of nanotherapy for HIV treatment and cure still face important obstacles. Despite improvements in formulation chemistry, achieving sufficient production scaling for the treatment of millions of individuals without compromising batch-to-batch and intra-batch variations remains challenging for most nanotherapeutic designs. Moreover, given the fact that most HIV-infected individuals are based in low-income countries, keeping production costs at a minimum is highly relevant. Finally, addressing the safety in case of adverse events and enable rapid drug discontinuation is a key.

## Figures and Tables

**Table 1:** FDA-approved pharmaceuticals for HIV treatment of HIV infection.

Drug Class	Name (Acronym)	Plasma T_½_ in Humans (h)	Absolute Bioavailability (F_abs_)
Nucleoside/nucleotide reverse transcriptase inhibitor (NRTIs)	Zidovudine (ZDV/AZT) [13]	1.2	60–70%
	Didanosine (DDI) [14]	1.5	25–43%
	Stavudine (d4T) [15]	1.6	82–99%
	Lamivudine (3TC) [16,17]	5.4	86–88%
	Abacavir (ABC) [18]	1.3	83%
	Tenofovir disoproxil fumarate (TDF) [19]	18.3	25% in fasting, increased with food
	Tenofovir alafenamide (TAF) [20]	51.3	n/a
	Emtricitabine (FTC) [21]	4.8	~100%
Non-nucleoside reverse transcriptase inhibitors (NNRTIs)	Efavirenz (EFV) [22]	37.7	~100%
	Nevirapine (NVP) [23,24,25,26]	21.5	90–93%
	Extended-release NVP [27]	45	n/a
	Etravirine (ETR) [28,29]	30–50	n/a
	Rilpivirine (RPV) [30]	48	n/a
Protease inhibitors (PIs)	Saquinavir (SQV) [31]	3.6	4–12%
	Ritonavir (RTV) [32]	3.5	60%
	Indinavir (IDV) [33]	1.8	~100%
	Nelfinavir (NFV) [31,34]	4.3	~100%
	Lopinavir (LPV) [35]	5–6	n/a
	Lopinavir (LPV) oral pellets	5–6	n/a
	Fosamprenavir (FPV) [36]	4.8	n/a
	Atazanavir (ATV) [37]	7.5	Low
	Tipranavir (TPV) [38]	2.6	n/a
	Darunavir (DRV) [39,40]	14.6	37% (w/o ritonavir), 82% (with ritonavir)
Fusion inhibitors	Enfurvirtide (T-20) [41]	2	n/a
Entry inhibitors	Maraviroc (MVC) [42]	23	23.1–33%
Integrase inhibitors (INIs)	Dolutegravir (DTG) [43,44]	13.5	87% (in monkeys)
	Elvitegravir (EVG) [45,46]	9.9	<25%
	Raltegravir (RAL) [47]	9.3	n/a
	Bictegravir (BIC) [48]	17.3	n/a

FDA-approved drugs for treatment of HIV infected are listed, including their biological target, year of approval, full name and acronym. Plasma half-lives after a single oral or intravenous dose are listed for each drug. The absolute bioavailability is listed where available and calculated based on the calculation: $F_{abs}=100\cdot \frac{AUC_{po}\cdot D_{iv}}{AUC_{iv}\cdot D_{po}}$. Note, the calculation of absolute bioavailability relies on data from studies done using intravenous administration, which are not available for many recent ARVs. (n/a =, not available). Adapted from [49,50].
